# Evaluation of Automated Hypnogram Analysis on Multi-Scored Polysomnographies

**DOI:** 10.3389/fdgth.2021.707589

**Published:** 2021-07-26

**Authors:** Dries Van der Plas, Johan Verbraecken, Marc Willemen, Wannes Meert, Jesse Davis

**Affiliations:** ^1^Onafhankelijke Software Groep (OSG bv), Micromed Group, Kontich, Belgium; ^2^Department of Computer Science, Leuven AI, KU Leuven, Leuven, Belgium; ^3^Faculty of Medicine and Health Sciences, University of Antwerp, Antwerp, Belgium; ^4^Multidisciplinary Sleep Disorders Centre, Antwerp University Hospital, Antwerp, Belgium; ^5^Department of Pulmonary Medicine, Antwerp University Hospital, Antwerp, Belgium

**Keywords:** hypnogram analysis, sleep stage scoring, model uncertainty, inter-rater reliability, machine learning, polysomnography

## Abstract

A new method for automated sleep stage scoring of polysomnographies is proposed that uses a random forest approach to model feature interactions and temporal effects. The model mostly relies on features based on the rules from the American Academy of Sleep Medicine, which allows medical experts to gain insights into the model. A common way to evaluate automated approaches to constructing hypnograms is to compare the one produced by the algorithm to an expert's hypnogram. However, given the same data, two expert annotators will construct (slightly) different hypnograms due to differing interpretations of the data or individual mistakes. A thorough evaluation of our method is performed on a multi-labeled dataset in which both the inter-rater variability as well as the prediction uncertainties are taken into account, leading to a new standard for the evaluation of automated sleep stage scoring algorithms. On all epochs, our model achieves an accuracy of 82.7%, which is only slightly lower than the inter-rater disagreement. When only considering the 63.3% of the epochs where both the experts and algorithm are certain, the model achieves an accuracy of 97.8%. Transition periods between sleep stages are identified and studied for the first time. Scoring guidelines for medical experts are provided to complement the certain predictions by scoring only a few epochs manually. This makes the proposed method highly time-efficient while guaranteeing a highly accurate final hypnogram.

## 1. Introduction

In polysomnographic (PSG) recordings, physiological signals like the electroencephalogram (EEG), the electromyogram (EMG), the electrooculogram (EOG), heart activity (ECG), and the patient's breathing pattern are measured over an entire night to assess sleep disorders such as sleep apnea or insomnia. The first step in making a final diagnosis is to construct the hypnogram, which is a leveled graph showing the sleep stage in function of time. Sleep stage scoring is often performed manually by clinical experts following rules determined by the American Academy of Sleep Medicine (AASM). By these rules, the PSG is divided into 30-s sequential windows starting from the beginning of the PSG. Each window, called an epoch, should be annotated by one out of five sleep-wake stages (W, N1, N2, N3, or R) (1).

Each of these sleep-wake stages has its own characteristics. The wakefulness stage (W) has in general a high EMG value, dominating alpha (8–13 Hz) and beta (14–35 Hz) waves in the EEG signal and can also be detected by the presence of eye blinks. The four remaining sleep stages are divided in two groups the REM stage R and the non-REM stages N1, N2, and N3. The former is characterized by rapid eye movements (REMs) in the EOG signal. The non-REM stage are divided in three categories from N1 (light sleep) to N3 (deep sleep). The correct category can be derived based on the presence of specific patterns in the data such as slow eye movements in N1, K-complexes and sleep spindles in N2 and slow EEG waves in N3. A complete overview can be found in the AASM guidelines (1).

Recently, multiple approaches have been proposed that apply machine learning to analyze the collected data and automatically construct a hypnogram. A wide range of classification techniques has been used such as deep learning (2, 3), support vector machines (4, 5), or random forests (6–9). Furthermore, the studies can be differentiated based on the considered physiological signals, with the majority focusing on EEG while others only use ECG or EOG. However, only a few approaches have attempted to combine all the information collected in a PSG (10). Overviews of these methods can be found in (10) and in (11). Most models are trained on either the raw signal data or on a complex set of features extracted from the time and frequency domain where commonly used techniques include empirical mode decomposition (8), singular value decomposition (9) and wavelet-based features (7). These approaches have two drawbacks. First, the complexity of the feature set and models makes sleep lab technicians hesitant to use these methods in practice. Second, if the technicians report a specific violation of one of the AASM guidelines, it is not straightforward to know how to improve the model when using black-box models or complex features.

This paper proposes a three step approach to automated hypnogram construction. The first step constructs a set of base features that focus on ensuring interpretability. Some of them, such as the occurrence of rapid eye movements (REMs) or sleep spindles, are derived from the EEG, EOG, and EMG signals based on the AASM guidelines whereas others have a straightforward medical interpretation such as the heart rate and body position. The second step derives a new feature set by computing the conditional probability of each base feature given the sleep stage. Experts can still verify whether this new set of features is meaningful from a medical point of view. In the last step, a random forest classifier combines all features within an epoch, together with information from consecutive epochs to predict the sleep stage. This corresponds largely to the human way of scoring which increases a medical expert's confidence in the model. Moreover, this classifier allows estimating the posterior probabilities for each sleep stage and hence, the uncertainty of the prediction.

Validating automatic hypnogram analyses is often performed by comparing the predicted hypnogram to an expert's scoring. The confusion matrix, as well as derived values such as the accuracy, kappa value or F1-value, can be reported (11). However, studies have shown that the inter-rater agreement is often below 85% as experts may make mistakes or have differing interpretations (12, 13). Alas, current studies do not consider inter-rater variability when evaluating algorithms for automating hynogram construction.

In this paper, a traditional evaluation of our proposed algorithm on a large single-scored dataset is performed. Afterwards, the influence of the inter-rater variability on the performance evaluation is analyzed on multi-labeled PSGs. Stephansen et al. proposed a hypnodensity visualization that shows the mutual disagreement between human experts (14). This can then be compared visually to the posterior probabilities of the sleep stages. However, the paper lacks an extensive quantitative evaluation and does not specify how to handle uncertain predictions. In contrast, this paper performs an evaluation that considers both the inter-rater variability and the prediction uncertainty. Moreover, it proposes guidelines to help medical experts interpret the algorithm's results in a manner that will allow them to efficiently verify uncertain predictions by providing a minimal number of manual labels.

The main contributions of this work are

1. A new method to predict the hypnogram that identifies epochs where the algorithm is unsure about its prediction.
2. Guidelines for medical experts to efficiently verify the uncertain predictions.
3. A new state-of-the art quantitative evaluation of the algorithm which takes into account both the prediction uncertainty as well as the uncertainty of the expert's labels.

## 2. Materials and Methods

This section consists out of three parts. In the first part, the used datasets are described. The second section contains a description of the proposed model for automated sleep stage scoring. The last section contains information on the prediction certainties and how to use them in a real-life medical setting.

### 2.1. Data

Two PSG datasets are used: a single-scored and a multi-scored dataset. The single-scored dataset contains 5884 PSGs from 10 medical centers in Belgium, the Netherlands and Austria where each PSG is scored by one expert. Patients were monitored in the centers on suspicion of both breathing and non-breathing sleep disorders such as apnea, insomnia, restless legs syndrome or narcolepsy. The average age of the patients was 49.1 years (std. 15.8). Patients under the age of 1 were excluded, as well as multi-sleep latency tests and PSGs lasting less than 1 h. Patients were not excluded based on their pathologies or medication use such that the dataset is representative for the entire population of patients examined in the medical centers.

The dataset is divided into a training, validation and test set such that there are an equal number of PSGs in each set. The training set is used to learn the parameters of the emission models and the random forest classifier discussed in section 2.2. The validation set was used to select appropriate hyperparameters for the model. Finally, a large, independent test set containing 2,461,303 labeled epochs remains, which is used in the evaluation discussed in section 3.

The multi-scored dataset contains only 50 PSGs (71,195 labeled observations) from a single center where each PSG is scored multiple times: 5 PSGs are scored by four clinical experts, 22 by five experts, 22 by six experts and the remaining 2 PSGs are scored by seven experts. Very few datasets exist that have been labeled by multiple due to time cost associated with collecting the annotations. The average age of the patients was 48.3 years (std. 17.5). The multi-scored dataset serves only as a test set to further evaluate the model learned from the single-scored training set. Hence, all results in this paper are validated on an independent dataset.

### 2.2. Automated Hypnogram Model

**Figure 1:**
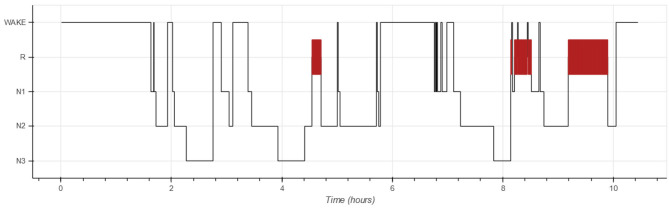
Example of a hypnogram scored by an expert.

Each PSG is partitioned into 30-s non-overlapping windows called epochs (1). The goal of automated hypnogram analyses is to predict the sleep stage for each epoch. The hypnogram itself is a graph, showing the sleep stage as a function of time in which epochs with sleep stage R are typically highlighted. Figure 1 shows an example of a hypnogram.

Our method to predict the hypnogram consists out of four steps. First, features based on the AASM guidelines (e.g., the number of REMs or sleep spindles) are extracted in each epoch. Second, the emissions, which are the conditional probabilities of the features given a certain sleep stage, are modeled. The emissions are a feature-construction step that makes the relation between the initial features and the sleep stages explicit. Third, these emissions are normalized. Fourth, the hypnogram is constructed combining information across multiple epochs. Next, each step will be further elaborated.

#### 2.2.1. Feature Extraction

The AASM describes rules to score the sleep stage in each epoch based on information from EEG, EOG, and EMG signals. The detection of the features described in the AASM, as well as some additional features is discussed in this section. Table 1 provides an overview of the considered features per signal type and also includes which sleep stages each features may be indicative of.

**Table 1:** Table summarizing all features per signal.

**Signal**	**Feature**	**Feature description**	**Indicating**
EEG	ORPW, ORPN1, ORPN2, ORPN3, ORPR	Sleep stage likeliness based on the EEG powers in the δ, θ, α and β bands, see (15)	W/N1 N2/N3/R
	Spindle	Number of trains of sinusoidal waves (11–16 Hz range) lasting more than 0.5 s.	N2
	Spindle density	Average number of spindles per epoch in a PSG	
EOG	REM	Number of conjugate, irregular, sharply peakedeye movements with an initial deflection <0.5 s.	W, R
	REM density	Average number of REMs per epoch in a PSG.	
	SEM	Number of conjugate, regular sinusoidal eye movements with initial deflection >0.5 s.	N1
	SEM density	Average number of SEMs per epoch in a PSG.	
EMG	EMG background	Average over all EMG signals after filtering the intentional movements.	W, R
ECG	Heart rate	Quantile of the average heart rate in the PSG.	W, N3*
SaO2	SaO2 average	Average oxygen saturation.	W, R*
	SaO2 variance	Variance of the oxygen saturation.	W*, N3*, R
Position	Position change	Indicator for a change in body position.	W
Sound	snores	Number of snores	W*

A description is given as well as the sleep stage in which the feature is expected to be high. Stages for which the feature is expected to be low are indicated with an *.

The dominant frequency of the EEG signal is probably the most important characteristic of a sleep stage. Therefore, the power of the EEG signal is calculated in four frequency bands (delta: 0–2.5 Hz, theta: 2.5–6.8 Hz, alpha: 6.8–14 Hz, beta: 14–35 Hz). Notice that the frequency bands deviate slightly from the definitions in the AASM guidelines. These four powers are combined in a single feature, the *Odds Ratio Product* (ORP), which is an indication for sleep stage W (15). ORP features are obtained for the other four sleep stages in an analogous manner.

*Sleep spindles* are trains of sinusoidal waves in the 11–16 Hz frequency range (often 12–14 Hz) that last more than 0.5 s (1). The sixth feature is the number of spindles in each epoch which is obtained using an unsupervised spindles detection method. The method first partitions the signal into segments based on zero-crossings. Next, a factor is derived for each segment based on its duration *d* and maximum amplitude *a* that indicates how likely it is that the segment is a part of a spindle. The wave's frequency *f* can be expressed as 1/2*d*. For each segment, a frequency score *s*_*f*_ is defined which is equal to 1 if the segment's frequency is between 12 and 14 Hz, and linearly decreases to 0 at frequencies of 10.5 and 16 Hz:


(1) $$\begin{array}{l} s_{f}\left(x\right)=\{\begin{array}{ll} \frac{2}{3}\left(x-10.5\right) & \text{if }10.5\leq x<12\\ 1 & \text{if }12\leq x<14\\ 1-\frac{1}{2}\left(x-14\right) & \text{if }14\leq x<16\\ 0 & \text{else.} \end{array} \end{array}$$

Similarly, an amplitude score *s*_*a*_, which peaks at 4 μ*V*, expresses whether the segment's amplitude is expected for a spindle:


(2) $$\begin{array}{l} s_{a}\left(x\right)=\{\begin{array}{ll} \frac{1}{4}x & \text{if }0\leq x<4\\ 1-\frac{1}{8}\left(x-4\right) & \text{if }4\leq x<12\\ 0 & \text{else} \end{array} \end{array}$$

The frequency of the spindle should be rather constant for multiple segments and hence, the variance of the frequency should be low. Combining all three conditions, a segment is likely to belong to a spindle if the spindle factor


(3) $$\begin{array}{l} \frac{\text{var}\left(\left\{f_{i}|j-5\leq i\leq j+5\right\}\right)}{s_{f}\left(f_{i}\right)s_{a}\left(a_{i}\right)} \end{array}$$

is low where *f*_*i*_ is the frequency and *a*_*i*_ is the amplitude in the i-th segment. Spindles are predicted for sequences of segments that have low spindle factors for at least 0.5 s.

As a seventh EEG feature, the *spindle density*, the average number of spindles per epoch, is computed for each PSG. This value might vary substantially among patients and hence, is needed to interpret whether a certain number of spindles is high for the examined patient. This will be explained in the next section on the emissions.

In addition to the seven EEG features, four features are derived from the EOG signal. These are the number of *slow eye movements* (SEMs) and the number of *rapid eye movements* (REMs), as well as their averages in the entire PSG which are calculated in an analogous manner as the spindle density. SEMs are sinusoidal movements where the initial deflection takes more than 0.5 s. These are typical in sleep stage N1. REMs are faster and more sharply peaked, and they suggest R sleep or a patient in sleep stage W who is looking around. The SleepRT software (OSG bv, Micromed Group, Kontich, Belgium) is able to detect both REMs and SEMs as half waves satisfying particular conditions. These REM and SEM densities are used since they can reveal important patient-dependent effects: An increased REM density is, for example, common for depressed patients while antidepressant drugs may suppress REMs (16, 17).

Only one EMG feature is derived which is the *average background EMG* over all chin and leg sensors. Hereto, a bandstop filter is used to filter out electric hum. Afterwards, a bandpass filter is applied and the morphological opening is calculated for each epoch to get the background EMG, which is the EMG expressing the muscle tonus without the tension related to activities such as swallowing, talking or other body motions (18). Median centering over the entire PSG is used such that epochs with background EMG higher than 1 point out an elevated EMG. This feature is often high in stage W and low in stage R.

Besides EEG, EOG, and EMG signals, most PSGs contain additional information that, while not used by the AASM manual for sleep scoring, can nevertheless be informative for constructing the hypnogram. They will now be discussed briefly.

The *heart rate* decreases from wakefulness to deep sleep but increases during R sleep (19). Because heart rate is highly person dependent, the quantile of the epoch's heart rate in the entire PSG is used as a feature instead of the heart rate itself.

A sensor measuring the body position is used to indicate five labels (standing, belly, back, left, and right). Although standing seems a strong indication that the patient is awake, this is in our data often not the case due to measurement errors. However, if a transition from lying down to standing up is observed, this is a more accurate predictor for sleep stage W. A binary feature indicating the epochs with such a *transition in body position* is used.

During R sleep, the strength of some of the breathing-related muscles decreases (20). This may be the reason why the average oxygen saturation is lower in R sleep than in non-R sleep. This hypoxia can occur due to apnea or hypopnea which also result in high variance of the oxygen saturation. On the other hand, when the subject is awake, the oxygen saturation level is rather high and constant (20). The *mean and variance of the oxygen saturation* level are therefore very informative for the sleep stage and are also used. Finally, the presence of *snores* can be used which are determined by analyses of the microphone signal.

#### 2.2.2. Emission Models

This section describes how to transform the features based on the AASM guidelines into a new feature set called emissions, representing the conditional probabilities of the feature values given the sleep stage *s*. This intermediate step helps make relation between the initial features and sleep stages clear. Furthermore, linking the initial features to the sleep stages allows investigating which features are informative on their own and which features should be combined, whether extra preprocessing is useful or how to deal with the patient-dependency of some features. Since experts can interpret these emission probabilities, they can provide advice to help solve these challenges. Moreover, experts can verify that the obtained emissions are meaningful from a medical point of view.

Let **f** = {*f*_1_, …, *f*_*m*_} be the set all *m* feature values in an epoch. The emissions *e*_*i, s*_ are defined for a subset *x* of **f** and are given by


(4) $$\begin{array}{l} e_{i,s}\left(x\right)=\frac{1}{c_{x}}p\left(x|s\right) \end{array}$$

with *p* a probability density function and *c*_*x*_ a normalization constant. The subset *x* often contains only one feature such as for the heart rate or EMG background. For sleep spindles, REMs and SEMs, the number of events is modeled simultaneously with the density value which allows interpreting the number of events per patient. Finally, the mean and average oxygen saturation are modeled together since their interaction is important to determine the sleep stage, an insight which is based on medical knowledge provided by experts.

This section discusses estimating *p*(*x*|*s*) while the subsequent section discusses how to normalize these probabilities. The emission models are time independent: the probability of observing a set of feature values given a sleep stage does not depend on the position of the epoch in the PSG. The AASM guidelines also makes this assumption.

The number of snores and the indicator for body position change are discrete features such that the probability density function in Equation (4) is replaced by a probability mass function. The maximum likelihood estimate of this function is equal to the normalized histogram of the feature values in the training data. The number of REMs, SEMs, and spindles are also discrete but highly patient-dependent. Therefore, they need to be handled differently in order to avoid weakening the relationship between the feature and the emission values, which would in turn make it more difficult to distinguish among sleep stages.

This can be solved by modeling the number of REMs *x* itself and the REM density ρ simultaneously. The density function for the REM distribution is


(5) $$\begin{array}{l} p\left(x,\rho |s\right)=\frac{1}{c}\frac{a_{x,s}}{a_{x,s}+\rho^{-b_{x,s}}}\text{ for }x\in \left\{0,\ldots ,3\right\},s\in S \end{array}$$

with parameters *a*_*x, s*_ and *b*_*x, s*_, normalization constant *c* and S={W,N1,N2,N3,R} the set of sleep stages. The number of REMs is truncated at 3 to guarantee a stable result. The parameters can be optimized using a maximum likelihood approach on a training set. Similarly, the appropriate density function for the number of SEMs is


(6) $$\begin{array}{l} p\left(x,\rho |s\right)=\frac{1}{c}e^{a_{x,s}}e^{\rho b_{x,s}}\text{ for }x\in \left\{0,1\right\},s\in S. \end{array}$$

The density of the number of spindles is given by a negative binomial distribution with mean equal to *aρ*^*b*^ and variance equal to *aρ*^*b*^ + *cρ*^*d*^. The four model parameters can again be optimized based on a training set.

The EMG background is a continuous feature of which the distribution can be fitted well with


(7) $$\begin{array}{l} p\left(x|s\right)=\{\begin{array}{ll} \frac{x^{a_{s}}}{c} & \text{if }x<1\\ \frac{x^{-b_{s}}}{c} & \text{if }x\geq 1 \end{array} \end{array}$$

with


(8) $$\begin{array}{l} c=\frac{1}{a_{s}+1}+\frac{1}{1-b_{s}}, \end{array}$$

*a*_*s*_ > −1 and *b*_*s*_ < 1 to guarantee normalization. The density peaks at 1, which corresponds to the median value, and decreases to zero by a power law.

The two remaining distributions are the joint distribution of the mean and variance of the oxygen saturation and the joint distribution of the heart rate and heart rate change. Both are two-dimensional and continuous which makes it hard to fit a parametric density model. Different non-parametric models can be used such as kernel density estimators.

The set of emissions contains 60 features for each epoch which can all be interpreted by human experts as each one corresponds to a feature-stage pair in which high values correspond to a posterior distribution which is higher than the prior. Take for example an EMG background value which is twice that high than the median EMG. The emission related to the stage W is then equal to 0.32 which is much higher than the R-emission which is only 0.05 stating that it is more likely to observe a high EMG when the patient is awake. This is a very intuitive and easy to verify statement for medical experts. In a similar way, it can, for example, be verified that a lot of spindles lead to a higher N2 emission or that the presence of SEMs increases the N1 emission.

#### 2.2.3. Normalization

The magnitude of the conditional probabilities *p*(*x*|*s*) is highly dependent on *p*(*x*). Therefore, low values indicate that the observed value *x* is rare instead of being informative about how likely a sleep stage is. The normalization constant *c*_*x*_ in Equation (4) is set to *p*(*x*) to filter out this effect. As a consequence, it holds for all *x* that.


(9) $$\begin{array}{l} \underset{s\in S}{\sum }e_{i,s}\left(x\right)=1. \end{array}$$

#### 2.2.4. Sleep Stage Classification

Given a training set $T=\{\left(x_{i},s_{i}\right)$ for *i* ∈ 0, …, *n*} with feature vectors *x*_*i*_ and sleep stages *s*_*i*_, a random forest (RF) classifier is learned to predict *s* from *x*. An RF is an ensemble of decision trees, where each tree is learned on a bootstrap sample of T (i.e., by sampling *n* examples with replacement from T). Each decision tree recursively partitions the sample space into cells until a stopping criterion is met. Let $C_{x}$ be the cell containing observation *x*, then the predicted class probability *p*_*s*_(*x*) of *x* for class *s* is equal to the fraction of training instances of class *s* in $C_{x}$:


(10) $$\begin{array}{l} p_{s}\left(x\right)=\frac{\left|\left\{\left(x_{i},s_{i}\right)\in T|x_{i}\in C_{x},s_{i}=s\right\}\right|}{\left|\left\{\left(x_{i},s_{i}\right)\in T|x_{i}\in C_{x}\right\}\right|}. \end{array}$$

Averaging the predictions made by each tree in the ensemble yields the final predicted class probabilities of the random forest. The predicted sleep stage corresponds to the stage with highest predicted probability:


(11) $$\begin{array}{l} \hat{s}=\underset{s\in S}{\mathrm{argmax}}p_{s}\left(x\right) \end{array}$$

For the automated hypnogram analysis, only considering the vector of emissions $e_{t}=\left[e_{i,s}|\forall i,\forall s\in S\right]$ as the input features to the random forest is insufficient. First, the AASM states that the sleep stage in some epochs depend on the previous epoch. Second, basing the prediction on data of a single epoch would make the prediction very sensitive to noise. Therefore, two extra vectors are added as input to the random forest: the forward probabilities ***α***_*t*−1_ and the backward probabilities ***β***_*t*+1_. These contain respectively estimates of the sleep stage distribution in the previous epoch based on the information up until that epoch; and estimates of the sleep stage distribution in the next epoch based on the information from that epoch onward. Both are calculated iteratively.

The forward probability α_*t*_(*s*_*t*_) is the probability of sleep stage labels *s*_*t*_ in epoch *t* given all emissions up to epoch *t*:


(12) $$\begin{array}{l} \alpha_{t}\left(s_{t}\right)=P\left(s_{t}|e_{1:t}\right)=\underset{s_{t-1}\in S}{\sum }P\left(s_{t}|s_{t-1},e_{1:t}\right)P\left(s_{t-1}|e_{1:t}\right) \end{array}$$

with ***e***_1:*t*_ = {***e***_1_, …, ***e***_*t*_}. Assuming conditional independence


(13) $$\begin{array}{l} P\left(s_{t}|s_{t-1},e_{1:t}\right)=P\left(s_{t}|s_{t-1},e_{t}\right) \end{array}$$

and assuming that each sleep stage is independent of future emissions


(14) $$\begin{array}{l} P\left(s_{t-1}|e_{1:t}\right)=P\left(s_{t-1}|e_{1:t-1}\right) \end{array}$$

it holds that


(15) $$\begin{array}{l} \alpha_{t}\left(s_{t}\right)=\underset{s_{t-1}\in S}{\sum }P\left(s_{t}|s_{t-1},e_{t}\right)\alpha_{t-1}\left(s_{t-1}\right) \end{array}$$

for *t* ∈ 1, …, *n*. The probabilities *P*(*s*_*t*_|*s*_*t*−1_, ***e***_*t*_) can be estimated analogous to Equation (10) with *x* = (*s*_*t*−1_, ***e***_*t*_). The forward probabilities $\alpha_{t}=\left[\alpha_{t}\left(s_{t}\right)|\forall s\in S\right]$ can then be calculated iteratively with α_0_(*s*) the probability of a PSG starting in stage *s*.

The vector of backward probabilities $\beta_{t}=\left[\beta_{t}\left(s_{t}\right)|\forall s\in S\right]$ can be calculated in an analogous manner by using only information from future epochs:


(16) $$\begin{array}{l} \beta_{t}\left(s_{t}\right)=P\left(s_{t}|e_{t:n}\right)=\underset{s_{t+1}\in S}{\sum }P\left(s_{t}|s_{t+1},e_{t}\right)\beta_{t+1}\left(s_{t+1}\right). \end{array}$$

The predicted sleep stages can now be obtained from the random forest classifier with input feature vector in epoch *t* equal to


(17) $$\begin{array}{l} x_{t}=\left[\alpha_{t-1},e_{t},\beta_{t+1}\right]. \end{array}$$

### 2.3. Prediction Certainties

Automated sleep stage scoring is a useful tool for clinical experts to speed up the analysis of PSGs. However, this analysis will never perform perfectly in all circumstances as the data is often noisy, sensors can fail or rare pathologies can be misinterpreted. In these cases, the analysis should indicate for which predictions it is uncertain and also list the possible sleep stages. Thus, the algorithm can alert the human expert who can then closely examine and label these epochs manually.

The predicted class probabilities obtained from the random forest in Equation (10) are a first indication of how certain the model is in its prediction. For example, the model is more certain if *p*_ŝ_(*x*) is much higher than the probabilities associated with all other classes whereas it is less certain if two classes have similar probabilities. To help the human experts to interpret these probabilities, they are transformed into three categories. The first contains the epochs where the prediction is certain and no manual check is needed. The second category indicates situations where the algorithm assigns similar probabilities to two sleep stages. In this case, both possible sleep stages are given along with their corresponding likelihoods. Practical scoring guidelines will be provided such that a human expert can annotate large periods of uncertainty by scoring only a few epochs. The third category identifies a small number of epochs where the algorithm is highly uncertain. This categorization allows obtaining an accurate hypnogram while only requiring experts to manually score a limited number of epochs.

In order to obtain these categories, the prediction probabilities are transformed using three steps: smoothing, exclusion of unlikely classes and normalization. Each of them will be discussed in more detail in the next paragraphs. This section will be concluded with the practical scoring guidelines to score the epochs in the second category in an optimal way.

#### 2.3.1. Smoothing

Transitions between sleep stages are often not sharply defined. For example, the dominating EEG frequency usually changes gradually and hence, the probability of going from one sleep stage to another should evolve gradually too. Therefore, smoothing seems to be an appropriate first step. However, an exception is when a patient suddenly wakes up for a short period, which can happen, for example, due to environmental noise, apnea or body motion. In these cases, sudden jumps in prediction probabilities should not be smoothed. Similarly, short transitions to N1 are possible when arousals appear. Therefore, the prediction certainties of sleep stage W and N1 are only smoothed if the reduction of the value is smaller than one third meaning that small fluctuations are smoothed while large increases in the W and N1 probabilities are not. For N2, N3, and R, sharp transitions are not expected, so smoothing is always performed. The smoothing is done by a moving average with a window size equal to 3 epochs. The transformation becomes:


(18) $$\begin{array}{l} p_{s}^{\prime }\left(x_{t}\right)=\{\begin{array}{ll} \frac{1}{3}\left(p_{s}\left(x_{t-1}\right)+p_{s}\left(x_{t}\right)+p_{s}\left(x_{t+1}\right)\right) & \text{if }s\in \left\{\text{N2, N3, R}\right\}\text{ or }\frac{p_{s}\left(x_{t}\right)}{p_{s}^{\prime }\left(x_{t}\right)}>\frac{2}{3}\\ p_{s}\left(x_{t}\right) & \text{else}. \end{array} \end{array}$$

#### 2.3.2. Exclude Low Scores

Low, non-zero prediction probabilities are very common when using random forests because a probability of 0 is only predicted if for every tree in the random forest, no training instances of a class fall in the cell of the partition. However, if the probability associated with a class is sufficiently small compared to the probability of another class, there is no doubt between these sleep stages and the prediction probability of the unlikely class can be set to zero. As a threshold, a ratio of 1/3 is taken, so if there is a class that is at least 3 times more likely, the probability of the class will be set to 0:


(19) $$\begin{array}{l} p_{s}^{\prime \prime }\left(x_{t}\right)=\{\begin{array}{ll} 0 & \text{if }\exists \tilde{s}\in S:3p_{s}^{\prime }\left(x_{t}\right)\leq p_{\tilde{s}}^{\prime }\left(x_{t}\right)\\ p_{s}^{\prime }\left(x_{t}\right) & \text{else}. \end{array} \end{array}$$

#### 2.3.3. Normalization

As a last step, the transformed prediction certainties are normalized such that they can be interpreted as probabilities:


(20) $$\begin{array}{l} p_{s}^{*}\left(x_{t}\right)=p_{s}^{\prime \prime \prime }\left(x_{t}\right)=\frac{p_{s}^{\prime \prime }\left(x_{t}\right)}{\sum_{\tilde{s}\in S}p_{\tilde{s}}^{\prime \prime }\left(x_{t}\right)}. \end{array}$$

This step concludes the transformation. For simplicity, the result after the transformations will be denoted as $p_{s}^{*}\left(x_{t}\right)$.

#### 2.3.4. Practical Scoring Guidelines

By design, the transformed class probabilities are zero for unlikely classes. This enables categorizing epochs by the prediction certainty based on the number of classes for which $p_{s}^{*}\left(x_{t}\right)$ is non-zero: if there is one class with $p_{s}^{*}\left(x_{t}\right)$ equal to 1, it is a certain prediction which should not be verified; if there are more than 2 classes with non-zero $p_{s}^{*}\left(x_{t}\right)$, the algorithm is not sure at all about its prediction and a manual scoring is necessary. The case where there are exactly 2 non-zero classes deserves more attention.

In this case, it can also be argued that all these epochs should be checked manually since there is doubt. However, this is often not the case because the value of $p_{s}^{*}\left(x_{t}\right)$ contains a lot of information which can be used to reduce the required manual effort. To see why, consider two common cases. The first common pattern is a transition between two stages, let say N2 and N3. Here, there is a period predicted as N2, followed by a period where the likelihood of N2 decreases while N3 increases followed by a period where N3 is predicted. While the period where the model is uncertain can be rather long, only the transition needs to be determined. The AASM rules determine that an epoch is N3 if at least in 20% of the epoch there is slow-wave activity. If such an epoch is observed, it can be assumed that all future epochs will be N3 too since $p_{N3}^{*}\left(x_{t}\right)$ is increasing. If the epoch does not meet the N3 criterion, it is N2 as are all previous epochs. Consequently, only a very limited number of epochs should be checked in order to label the entire transition period.

A second common pattern also starts in N2 followed by a period of doubt between N2 and N3 but ends with a period of N2. In this case, the experts can verify the epochs where the probability of N3 is maximal. If it is judged that this is still N2, the entire period can be labeled as N2. If these epochs are labeled as N3, the steps from the previous example can be repeated to find the transition point. Therefore, also this period of uncertainty can often be labeled entirely based on labeling a small number of epochs by exploiting the information obtained from the model. Real-life examples will be shown in the section on hypnodensity graphs.

#### 2.3.5. Expert's Uncertainty

Besides uncertainty on the algorithm's prediction, there is also uncertainty on the expert's label. A measure for this uncertainty can be obtained for epochs which are scored by multiple experts. Let *s*_*tj*_ be the label of rater *j* in epoch *t*. Define ρ_*s*_(*x*_*t*_) as the fraction of experts scoring epoch *t* as s:


(21) $$\begin{array}{l} \rho_{s}\left(x_{t}\right)=\frac{1}{m_{t}}\overset{m_{t}}{\underset{j=0}{\sum }}\delta_{s_{tj},s} \end{array}$$

with


(22) $$\begin{array}{l} \delta_{a,b}=\{\begin{array}{ll} 0 & \text{if }a\neq b,\\ 1 & \text{if }a=b \end{array} \end{array}$$

and *m*_*t*_ the number of raters who scored epoch *t*. This value can be interpreted such as *p*_*s*_(*x*_*t*_): a higher value indicates a higher certainty on class *s*. The maximum value over all sleep stages is denoted as $\rho_{\bar{s}}\left(x_{t}\right)$, in analogy to *p*_*ŝ*_(*x*_*t*_).

Disagreement between experts can have multiple causes, some of which are intrinsic to the data such as noise, others which are just due to human errors or misinterpretations. It is often difficult to make a distinction between these two categories. However, an expert's opinion which clearly deviates can be assumed to be individual mistakes. This is similar in spirit to the approach taken in Equation (19) which excluded unrealistic classes. There, it is assumed that if there is a stage which is at least three times more likely than another stage, the unlikely stage can be ignored. Therefore, Equations (19) and (20) are applied on ρ_*s*_(*x*_*t*_). The notation $\rho_{s}^{*}\left(x_{t}\right)$ denotes the transformed values. So, besides the uncertainty of the predicted label *p*_ŝ_(*x*_*t*_), the uncertainty of the expert's label $\rho_{\bar{s}}\left(x_{t}\right)$ can also be quantified for epochs which are scored multiple times. However, it should be taken into account that the number of labels is often rather small such that only a very rough estimate is obtained.

## 3. Results

A standard evaluation of an automated sleep stage model consist of a comparison of the automated hypnogram with a single expert's hypnogram. The results of this analysis and the shortcomings are discussed in section 3.1. From section 3.2 onward, the multi-scored dataset is used. First, the inter-rater reliability is calculated which gives a general indication on the trustworthiness of the expert's labels. The relation between *p*_*s*_(*x*_*t*_), ρ_*s*_(*x*_*t*_) and the accuracy will be investigated in the section 3.3. A visual comparison between the uncertainties by hypnodensity graphs will follow next. A numerical analysis of the accuracy based on the obtained uncertainties follows in the last section.

### 3.1. Single-Scored Dataset

A comparison of the automated hypnogram with a single expert's hypnogram is the most common evaluation method used in automatic sleep stage scoring. A similar analysis is done on the first dataset containing 1961 PSGs. The confusion matrix can be seen in Table 2. This matrix *C* contains in the cell $C_{s\tilde{s}}$ the percentage of epochs for which the expert indicates sleep stage *s* while the predicted sleep stage is $\tilde{s}$:


(23) $$\begin{array}{l} C_{s\tilde{s}}=\frac{1}{n}\overset{n}{\underset{t=0}{\sum }}\delta_{s_{t},s}\delta_{{\hat{s}}_{t},\tilde{s}} \end{array}$$

with *s*_*t*_ the expert's label in epoch *t* and ŝ_*t*_ the predicted sleep stage. The accuracy, the fraction of epochs for which the predicted sleep stage correspond to the expert's label, is equal to 80.5%. It is easily verified that this corresponds with the sum of the diagonal element in the confusion matrix. Almost half of the mistakes are confusions between N1 and N2 (4.1%) or between N2 and N3 (5.0%). Also a W-N1 mistake seems to appear regularly (3.0%). From the row and column totals, showing the distribution over the sleep stages for the true and predicted labels, it seems that N2 is predicted 3.7% too often, while N1 is under predicted by 3.5%. Cohen's kappa is equal to 0.734 which is often seen as substantial (21). However, from this dataset, it is impossible to determine which fraction of this disagreement is caused by incorrect predictions and which fraction is due to mistakes from the experts, or whether there are epochs for which multiple sleep stages are acceptable, depending on the expert.

**Table 2:** Confusion matrix for the single-scored dataset, including row and column totals, comparing the experts' sleep stages to the predicted sleep stages.

		**Algorithm**					
		**W**	**N1**	**N2**	**N3**	**R**	
**Expert**	**W**	19.5	1.0	1.0	0.0	0.4	21.9
	**N1**	2.0	2.8	3.0	0.0	0.9	8.7
	**N2**	0.9	1.1	33.8	2.0	1.3	39.1
	**N3**	0.1	0.0	3.5	12.3	0.0	15.9
	**R**	0.4	0.3	1.5	0.0	12.1	14.3
		22.9	5.2	42.8	14.3	14.7	

### 3.2. Inter-rater Reliability

Let *n* be the number of epochs and let *s*_*tj*_ be the label of scorer *j* in epoch *t*. Denoting the number of experts which labeled epoch *t* by *m*_*t*_, a confusion matrix between two or more experts can be calculated by taking the average pairwise agreement between the *m*_*t*_ experts in each epoch:


(24) Css~=1n∑t=0n1(mt2)∑j≠kδstj,sδstk,s~.

Notice that this is a weighted confusion matrix with weights chosen such that each epoch is equally important, regardless of the number of human annotators. Since the order of the experts is unimportant, $C_{s\tilde{s}}$ and $C_{\tilde{s}s}$ can be combined in one cell. The result for the multi-labeled dataset is shown in Table 3.

**Table 3:** Percentage of epochs per sleep stage in a weighted pairwise comparison between experts.

	**W**	**N1**	**N2**	**N3**	**R**
**W**	15.2	2.4	1.0	0.0	0.3
**N1**		4.0	4.5	0.0	1.4
**N2**			38.1	4.7	1.4
**N3**				12.7	0.0
**R**					14.1

The inter-rater reliability (IRR) is a general measure for the similarity between the labels given by human experts and is defined as the fraction of epochs in which two human experts agree. Hence, it is comparable to the accuracy when comparing automated labels with expert's labels. Therefore, the IRR can be calculated as


(25) $$\begin{array}{l} IRR=\underset{s\in S}{\sum }C_{ss}. \end{array}$$

This value is 84.1%, which corresponds with values found in literature. However, all experts are from the same hospital. Therefore, it can be expected that the cross-center IRR could even be lower.

The IRR can now be compared to the accuracy on this multi-scored dataset. Again, the weights of the confusion matrix are chosen such that every epoch is equally important:


(26) $$\begin{array}{l} C_{s\tilde{s}}=\frac{1}{n}\overset{n}{\underset{t=0}{\sum }}\frac{1}{m_{t}}\overset{m_{t}}{\underset{j=0}{\sum }}\delta_{s_{tj},s}\delta_{{\hat{s}}_{t},\tilde{s}}. \end{array}$$

The confusion matrix is shown in Table 4. The accuracy is 82.7% which is only 1.4% lower than the mutual agreement between human experts. Therefore, the algorithm is only slightly outperformed by human experts. Moreover, the same types of mistakes is made which are mostly in between W-N1, N1-N2, and N2-N3 suggesting that a large part of the disagreement with the automated prediction is due to mutual disagreement between experts.

**Table 4:** Weighted confusion matrix of experts' labels compared to algorithm's prediction.

		**Algorithm**					
		**W**	**N1**	**N2**	**N3**	**R**	
**Expert**	**W**	14.7	1.0	1.0	0.0	0.5	17.2
	**N1**	1.4	2.5	3.5	0.0	0.8	8.2
	**N2**	0.6	0.7	39.4	2.3	0.9	43.9
	**N3**	0.0	0.0	2.8	12.2	0.0	15.0
	**R**	0.2	0.2	1.3	0.0	13.9	15.6
		16.9	4.4	48.0	14.3	16.1	

### 3.3. Comparison Uncertainties

**Figure 2:**
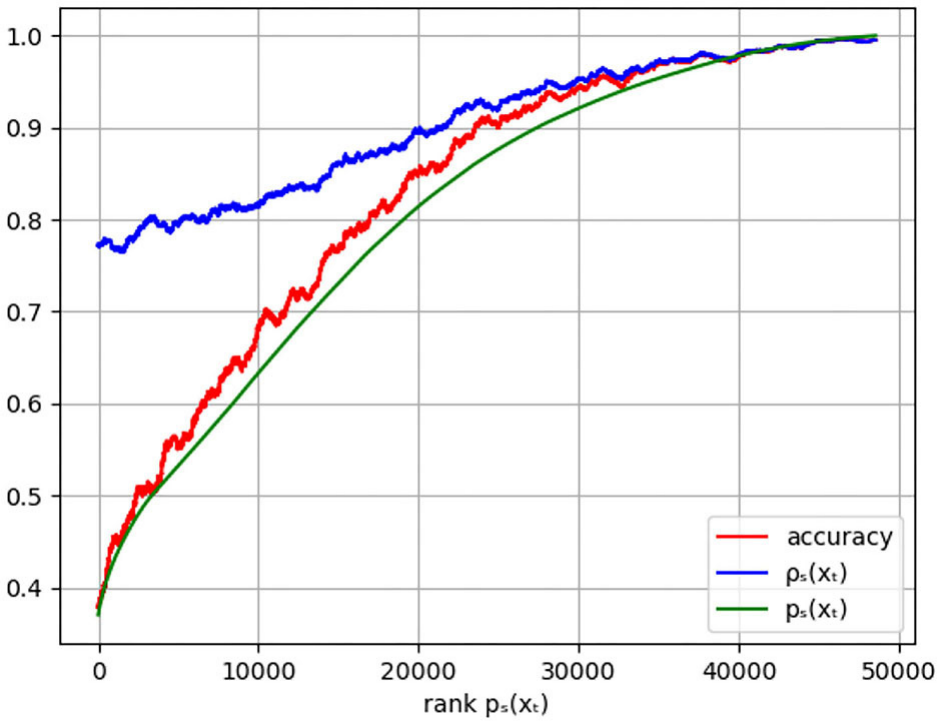
Plot of the moving average of the accuracy (red), the experts' certainty $\rho_{\bar{s}}\left(x_{t}\right)$ (blue), and the model certainty *p*_ŝ_(*x*_*t*_) (green) in function of the rank of *p*_ŝ_(*x*_*t*_). The accuracy is given by the weighted agreement between the predicted sleep stage and the experts' labels with weights such that every epoch contributes equally, independent of the number of experts' labels.

In the previous paragraph, the predicted sleep stage was compared to the experts' sleep stages. In this section, the relation between the prediction certainty *p*_ŝ_(*x*) and the certainty of the experts' labels $\rho_{\bar{s}}\left(x\right)$ is investigated. Figure 2 shows the moving average with window size 1,000 of both uncertainties in function of the rank of *p*_ŝ_(*x*) revealing that low prediction certainties correspond to epochs where the experts' labels are less certain. Hence, epochs with low prediction confidence might be hard to score for human experts too. This can, for example, happen for data that contains a lot of noise. On the other hand, for epochs where the model is rather confident, $\rho_{\bar{s}}\left(x\right)$ is high as well indicating agreement among the experts.

The same figure shows the moving average of the accuracy. As desired, high prediction certainty corresponds to high accuracy. It is observed that the prediction certainties are an underestimation of the actual accuracy, calibration techniques can be used to remove this difference. However, as discussed, the ratio's between the predicted class probabilities of all sleep stages turns out to be more informative than the prediction certainty alone.

### 3.4. Hypnodensity Graphs

**Figure 3:**
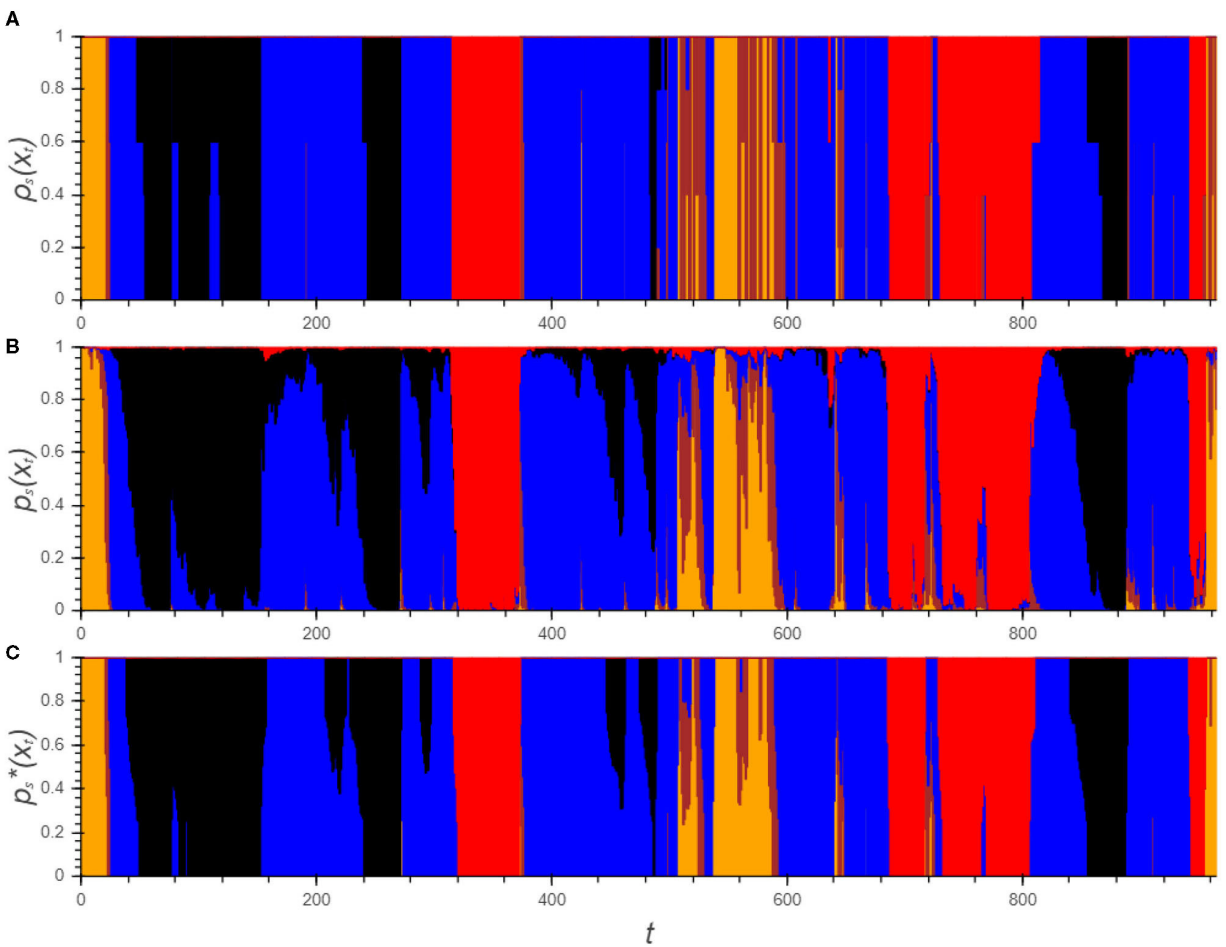
Hypnodensity graphs showing the experts' labels **(A)** as well as the algorithm's posterior class probabilities before **(B)** and after the transformations **(C)**. Each color represents a sleep stage (W: yellow, N1: brown, N2: blue, N3: black, R: red).

A hypnodensity graph is a fuzzy hypnogram in which sleep stages are visualized by stacked color bars with height equal to the probability of the sleep stage (14). For the automatic analysis, both *p*_*s*_(*x*_*t*_) and $p_{s}^{*}\left(x_{t}\right)$ can be is visualized. In a similar way, $\rho_{s}^{*}\left(x_{t}\right)$ can be used to indicate the agreement between the experts. A comparison between all three hypnodensities for one PSG is visualized in Figure 3 from which the similarity is immediately obvious.

Although the plot of *p*_*s*_(*x*_*t*_) corresponds largely to the hypnodensity of the experts, the drawbacks which were discussed in section 2.3 are clearly visible: First of all the signal is rather rough. Moreover, there are a lot of negligible effects visible such as the short bumps in which *p*_*W*_(*x*_*t*_) and *p*_*N*1_(*x*_*t*_) are elevated or *p*_*R*_(*x*_*t*_) which is only a few percent for a large part of the PSG. These patterns do not have any influence on the final prediction of the sleep stage and can make the interpretation of the graph confusing. After all, it is not straightforward how to use this information.

On the other hand, the graph of $p_{s}^{*}\left(x_{t}\right)$ clearly indicates for each epoch the possible sleep stages from which it is easy for experts to obtain a final hypnogram. As already pointed out, three main categories can be distinguished. For the majority of the epochs, only one sleep stage remains. Between epoch 200 and 260, there are three periods where there is uncertainty between N2 and N3: in the first and third, there is doubt between the stages but the algorithm predicts no transition to N3. All experts agree that both periods should be N2. Using the practical guidelines, only one epoch in each period should be scored to reach this insight. The second period of doubt is a transition from N2 to N3 in which the transition point should be determined. The expert's hypnodensity indicates that this choice is expert dependent. However, each expert can determine this point based on his own insight. The last category of epochs, in which the algorithm is unsure, can be observed in between epoch 500 and 550. These periods can be checked manually but the periods are rather short and it is therefore not an issue.

### 3.5. Accuracy Analysis

For both the algorithm and the experts, three categories are defined that correspond to the level of certainty of the label. Now, a comparison between both can be made. Table 5 gives an overview of the results. For the majority of the epochs (63.3%), both the algorithm and experts are certain, and hence only indicate one sleep stage. Of course, it is desired that the predicted sleep stage corresponds to the expert's consensus. This is the case for 97.8% of the epochs, so clear mistakes are made on just over 2% of the epochs. This is substantially better than the initial confusion matrix suggested and hence, demonstrates the need to perform an evaluation on a multi-scored dataset.

**Table 5:** Accuracy and fraction of epoch per category.

**Experts**	**Model**	**Fraction epochs**	**Accuracy**
Certain	Certain	63.3	97.8
Certain	Doubt	19.1	95.0
Doubt	Certain	4.7	91.5
Doubt	Doubt	7.8	84.9
Uncertain		0.7	
Certain/doubt	Uncertain	4.3	

*The calculation of the accuracy measure is slightly modified for each category as described in section 3.5*.

The second most common group contains epochs where the experts are unanimous but the algorithm cannot decide between two sleep stages. Here, a traditional accuracy measure would be rather low (71.9%), as the model confidence indicates. However, in this case it is mainly important that the experts can make the correct decision based on the provided information. Hence, it is required that one of the two suggested sleep stages corresponds to the expert's consensus. This is true for 95.0% of the epochs. For simplicity, this measure is also called accuracy in Table 5. It can be argued that the algorithm is unnecessary uncertain about its prediction. However, the categories are rather arbitrary and that the number of labels is limited. Therefore, one expert's label can change the category of an epoch.

If the experts offer two different opinions about the sleep stage of an epoch, a prediction may be seen as accurate if it corresponds to one of these opinions. This is the case for 91.5% of the epochs that were predicted with high certainty. For the epochs in which both the expert and the algorithm doubt between two sleep stages, for 84.9% of the epochs they doubt between the same stages. This is typical behavior when changing between sleep stages, especially between N2 and N3, since this transition is often rather gradual.

The last group of epochs are those where the algorithm cannot determine the sleep stage (4.3% of the epochs) or where the experts completely disagree (0.7% of the epochs). The first group should be scored manually, but since this is a rather small group, the manual analysis will still be fast. For the second group, it is impossible to determine whether the predicted sleep stage is correct, if such a stage already exists.

## 4. Discussion

A new method to perform automated sleep stage scoring is proposed that avoids constructing complex feature design, by designing a feature set that is largely based on the AASM rules. The relationship between the features and sleep stages is modeled, resulting in a new set of features, each indicating the likelihood of the features for a specific sleep stage. This feature set has as advantages that it is meaningful to medical experts and that gives insight in the optimal preprocessing as well as on how to deal with the patient-dependent nature of some features. A random forest performs the final sleep stage prediction by considering features extracted about the current epoch as well as the probabilities on the sleep stages in neighboring epochs. The feature importance, the fraction of decrease in impurity caused by a feature, shows the high influence of the neighboring epochs: 45.6% for the previous epoch and 35.6% for the next. The feature importance of the ORP emissions are between 1.6 and 3.0%, while these are 2.5% for REMs, 1.2% for sleep spindles and 0.8% for SEMs and for the background EMG. The emissions not related to AASM features have an even lower contribution.

Additionally to a sleep stage prediction, the random forest returns the predicted class probabilities *p*_*s*_(*x*_*t*_) which contain an indication of how certain the model is about its prediction. Transformations of *p*_*s*_(*x*_*t*_) are proposed to help medical experts interact with our method in a simple manner. Three levels for the prediction certainty are identified: epochs where the prediction certainty is high, epochs where the algorithm cannot decide between two stages, and epochs where the algorithm is not able to make an accurate prediction. The first set is by far the largest and contains 68.2% of the epochs. For these, no manual verification is necessary since the accuracy is high. The second category still contains 27.3% of the epochs. The transformed probabilities $p_{s}^{*}\left(x_{t}\right)$ can help human experts to score these periods with a small amount of manual effort. After all, large periods of the PSG can be identified based on a few labels. Transition periods between N2 and N3 are a typical example of this category. The remaining epochs which should be checked manually only comprise 4.3% of the PSG.

This methodology is entirely in line with the AASM position statement on artificial intelligence (AI) in sleep medicine (22) which states that the inclusion of machine learning should be approached cautiously and that manual sleep scoring of part of the PSG may still be necessary. Furthermore, this statement requires that AI programs for clinical use should be tested on an independent set of patients. This set should be sufficiently diverse to generalize to a heterogeneous patient population for which the software is intended. Finally, they demand that the program performs comparably to the agreement among experts.

Our paper meets these standards by performing two evaluations. The first is an extensive comparison of the automated hypnogram with an expert's hypnogram on a single-scored set of 1961 PSGs while the second evaluation is performed on 50 PSGs which are each scored by multiple experts. The single-scored set is representative of the patient population in Belgian and Dutch hospitals as the data comes from 10 different centers and is scored by a large group of different human experts. Moreover, our study considers a diverse patient population since there were no selection criteria used that are based on observed diseases or medication use.

The evaluation on this single-scored dataset leads to a confusion matrix from which the accuracy and κ-value, as well as the types of mistakes, can be derived. The obtained accuracy is equal to 80.5% with κ = 0.734. This is lower than some accuracies reported in literature: (3) and (14) report an accuracy of 87% using deep learning methods while (7) even reports an accuracy of 91.5% using a random forest classifier. However, in all these reported datasets, patients were excluded based on different criteria which makes it impossible to compare the accuracies across studies (23, 24). Moreover, the fact that certain types of patients were excluded from these publicly available datasets makes using them unsuitable.

Based on the first evaluation on the single-scored datasets, it is still impossible to estimate the influence of the inter-rater variability and hence, whether the algorithm is performing well compared to human experts. Therefore, the second evaluation method is introduced based on hypnograms scored by multiple experts. The inter-rater reliability on the multi-labeled dataset (84.1%) is barely higher than the comparison between the algorithm and the expert's hypnograms (82.7%). This suggests that the algorithm reaches an almost human accuracy but due to the inter-rater variability this is not clear from the accuracy value itself. Studying the inter-expert variability, the epochs can be categorized based on the level of agreement between the experts. An accuracy of 97.8% is reached considering only epochs in which both the experts and the algorithm are sure. This high level could never be obtained when validating on PSGs which are only labeled by one annotator. Also when the model is unsure about its prediction, the true sleep stage is still included in the proposed options 95% of the time. When the experts are not unanimous about the label, it is harder to define which results are acceptable. Future work might focus on evaluation methods in which experts can indicate more than one acceptable sleep stage. This will give a more realistic view on the performance of automated hypnogram models. Moreover, it can be a first step toward new scoring protocols in which gradual transitions between sleep stages are possible.

Our paper has the following three limitations: First, the detection of the features from the AASM manual are based on heuristics and are lacking a quantitative evaluation. Hence, the detection might be suboptimal. Second, although our model was evaluated on a diverse population, we were unable to verify that it performs equally well for all types of patients since no data about the patients' diagnoses was available. Third, a comparison with other models is hard since these models are evaluated on different datasets with other inclusion criteria. Moreover, their implementations are often not publicly available. These limitation might be investigated further in future work.

## Data Availability Statement

The datasets presented in this article are not readily available because no consent from the medical centers was provided to share their data with anyone beyond the authors. Requests to access the datasets should be directed dries.vanderplas@micromedgroup.com.

## Author Contributions

DV wrote the manuscript and performed the analyses. DV, JV, WM, MW, and JD contributed to the conception of the study and editing of the manuscript. MW contributed to the data collection. All authors approved the submitted version.

## Conflict of Interest

DV is an employee of OSG bv. The remaining authors declare that the research was conducted in the absence of any commercial or financial relationships that could be construed as a potential conflict of interest.

## Publisher's Note

All claims expressed in this article are solely those of the authors and do not necessarily represent those of their affiliated organizations, or those of the publisher, the editors and the reviewers. Any product that may be evaluated in this article, or claim that may be made by its manufacturer, is not guaranteed or endorsed by the publisher.
